# Supplementary material for: Multiple Stimuli-Responsive Conformational Exchanges of Biphen[3]arene Macrocycle
Source: Molecules. 2020 Dec 8;25(24):5780. doi: 10.3390/molecules25245780 (PMC7762528; doi:10.3390/molecules25245780)
Supplement: Supplementary file 1 [file molecules-25-05780-s001.pdf]

Electronic Supplementary Information

Multiple Stimuli-responsive Conformational Exchanges of Biphen[3]arene Macrocycle

Yiliang Wang[1]†, Liu-Pan Yang[2]†, Xiang Zhao[1], Lei Cui[1], Jian Li[1], Xueshun Jia[1], Jianhui Fang[1] and Chunju Li[1,3,*]

[1] School of Materials Science and Engineering, Center for Supramolecular Chemistry and Catalysis and Department of Chemistry, Shanghai University, Shanghai 200444, China; steven93916@126.com; zhaoxiang_shu@163.com; cuilei15@usst.edu.cn; lijian@shu.edu.cn; xsjia@mail.shu.edu.cn; jhfang@shu.edu.cn

[2] Academy for Advanced Interdisciplinary Studies, Southern University of Science and Technology, Xueyuan Blvd 1088, Shenzhen 518055, China; yanglp@sustech.edu.cn

[3] Key Laboratory of Inorganic-Organic Hybrid Functional Material Chemistry, Ministry of Education, Tianjin Key Laboratory of Structure and Performance for Functional Molecules, College of Chemistry, Tianjin Normal University, Tianjin 300387, China; cjli@shu.edu.cn

* Correspondence: cjli@shu.edu.cn; † these two authors contributed equally to this paper

1. Materials and Methods

2,2',4,4'-tetramethoxy biphen[3]arene (MeBP3) was synthesized according to our previous report[1]. Secondary ammonium guest **1**$^{+}$·BArF^{-} was synthesized according to the literature[2]. ^{1}H NMR (acquisition time = 3.17s, relaxation delay = 1.00s) and ^{13}C NMR (acquisition time = 1.09s, relaxation delay = 2.00s) spectra were recorded on a Bruker AV500 instrument. ^{1}H-^{13}C HMQC spectrum, ^{1}H-^{13}C HMBC spectrum and 2D NOESY NMR spectrum were recorded on a Bruker AVANCE III HD 600 MHz spectrometer. Variable-temperature ^{1}H NMR (acquisition time = 2.19s, relaxation delay = 5.00s) spectra were recorded on a JEOL JNM-ECZ400SL NMR spectrometer.

2. Copies of ^{1}H NMR and ^{13}C NMR spectra of MeBP3.

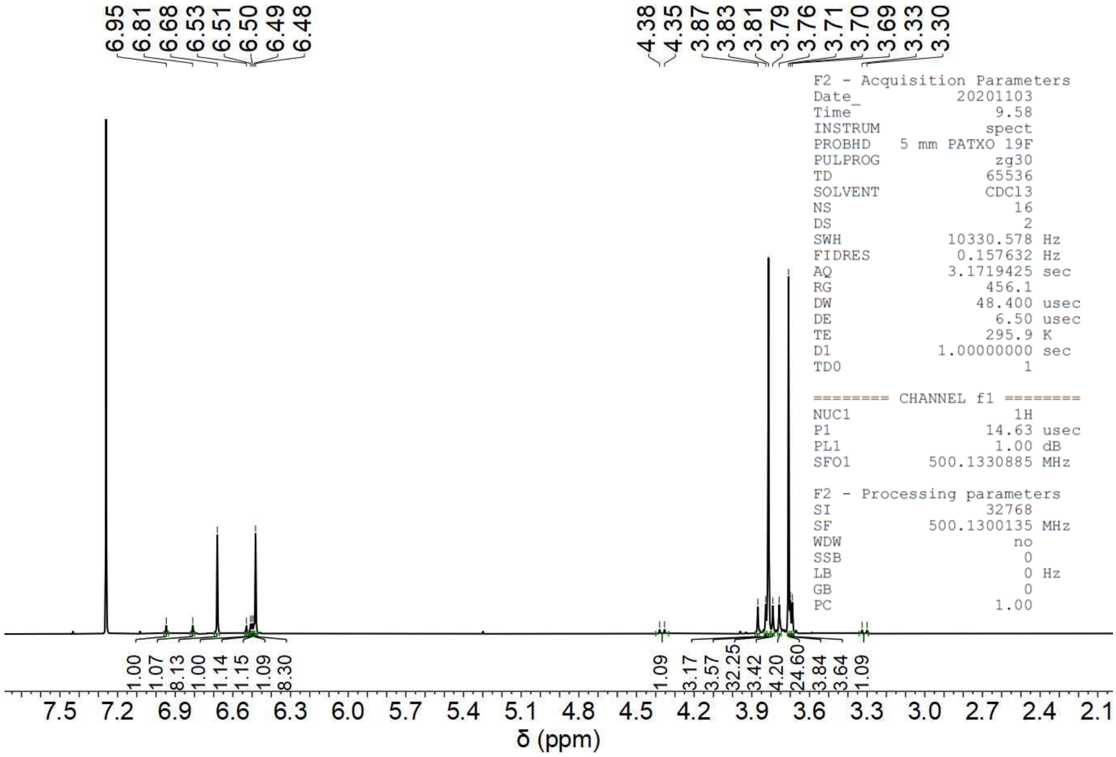

Figure S1 ^{1}H NMR spectrum (500 MHz, 25 °C, 2.0 mM) of MeBP3 in $CDCl_3$.

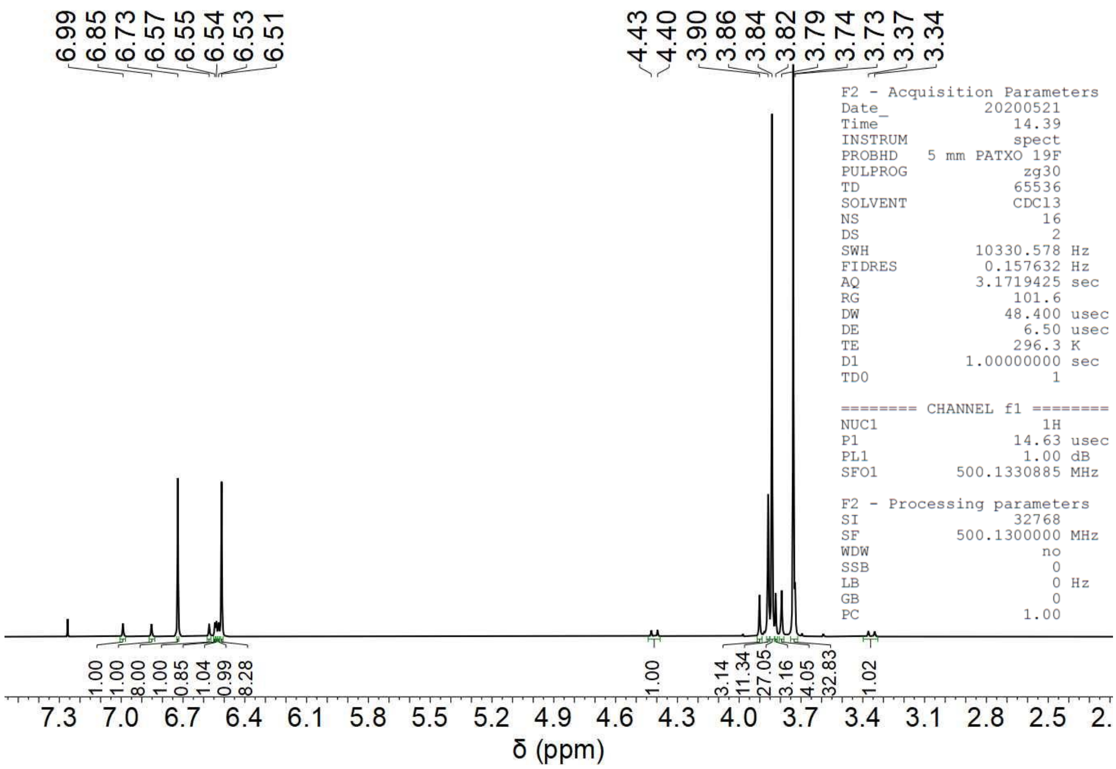

Figure S2 ^{1}H NMR spectrum (500 MHz, 25 °C, 100 mM) of MeBP3 in $CDCl_3$.

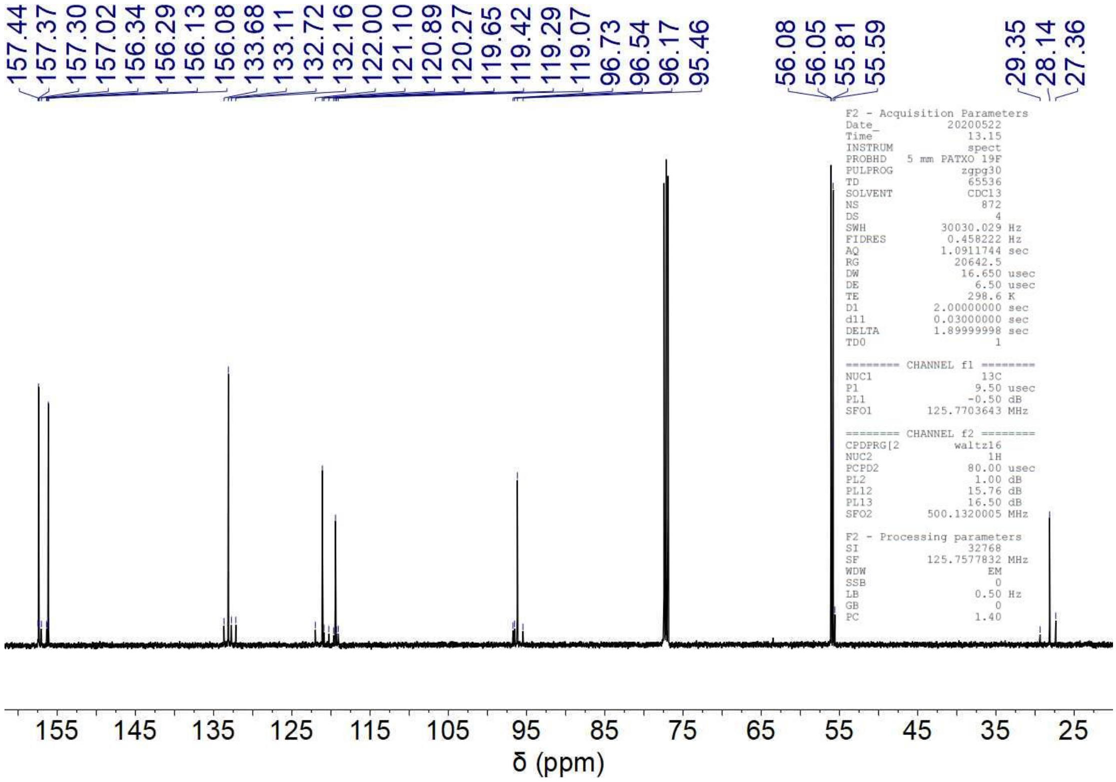

Figure S3 ^{13}C NMR spectrum (125 MHz, 25 °C, 100 mM) of MeBP3 in $CDCl_3$.

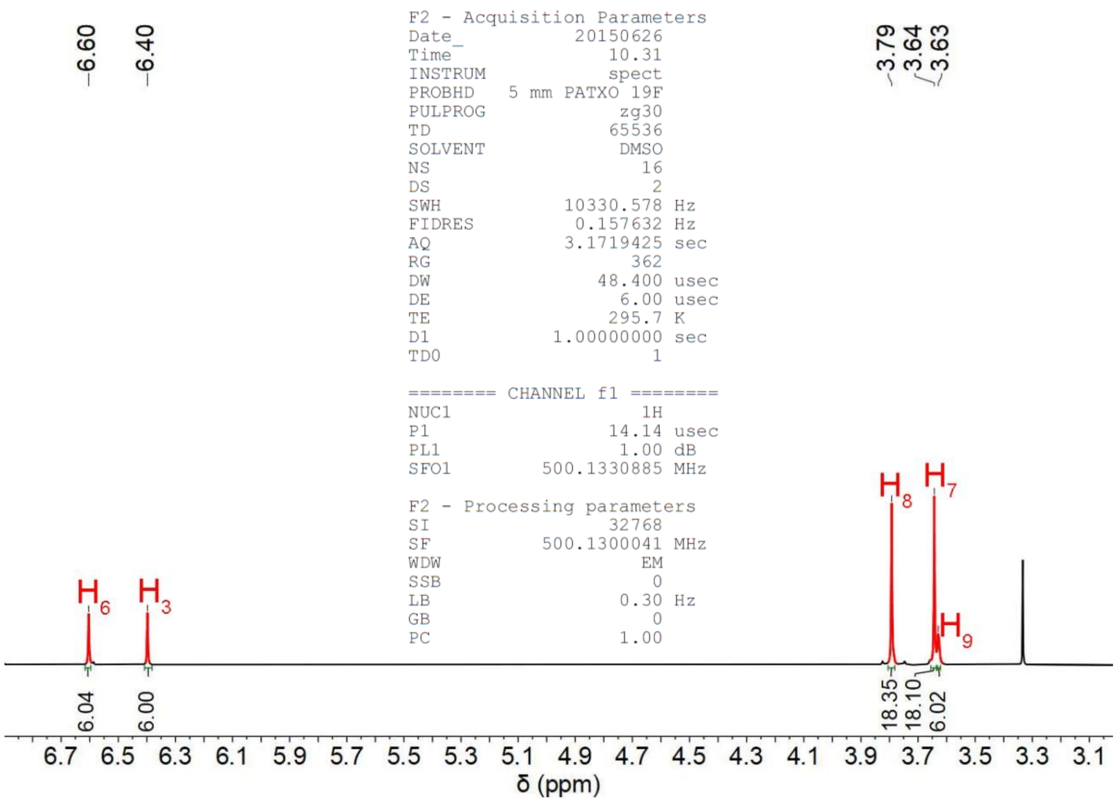

Figure S4 ^{1}H NMR spectrum (500 MHz, 25 °C, 2.0 mM) of MeBP3 in DMSO-d_6.

3. Structures assignment and ^{1}H-^{13}C HMQC/HMBC NMR & 2D NOESY NMR spectra.

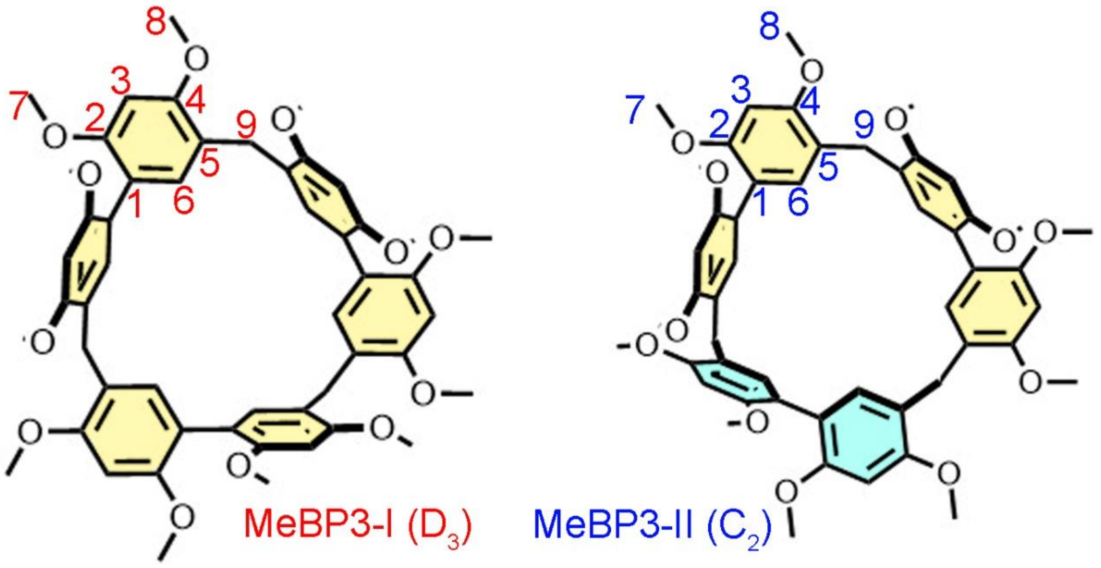

Figure S5 Chemical structures of two conformers of MeBP3.

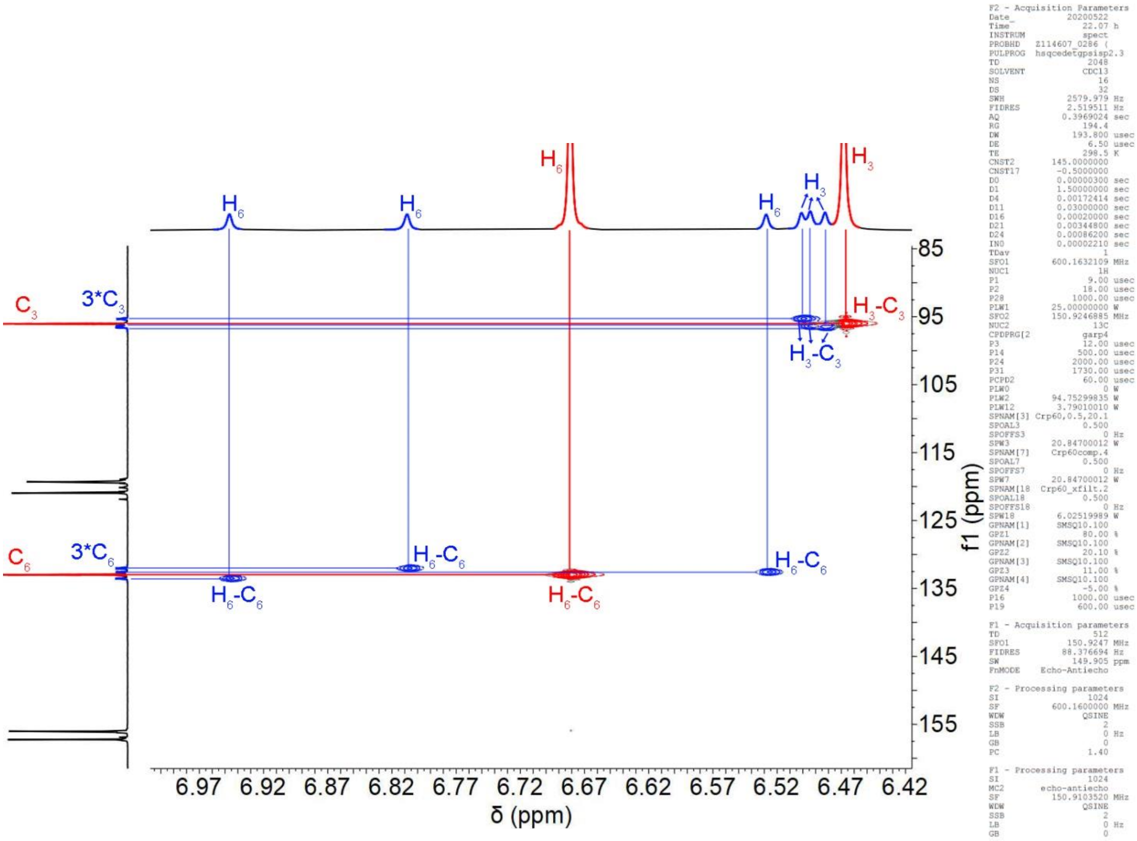

Figure S6 Partial ^{1}H-^{13}C HMQC NMR spectrum (600 MHz, 25 °C, 100 mM) of MeBP3 in $CDCl_3$ (aromatic regions).

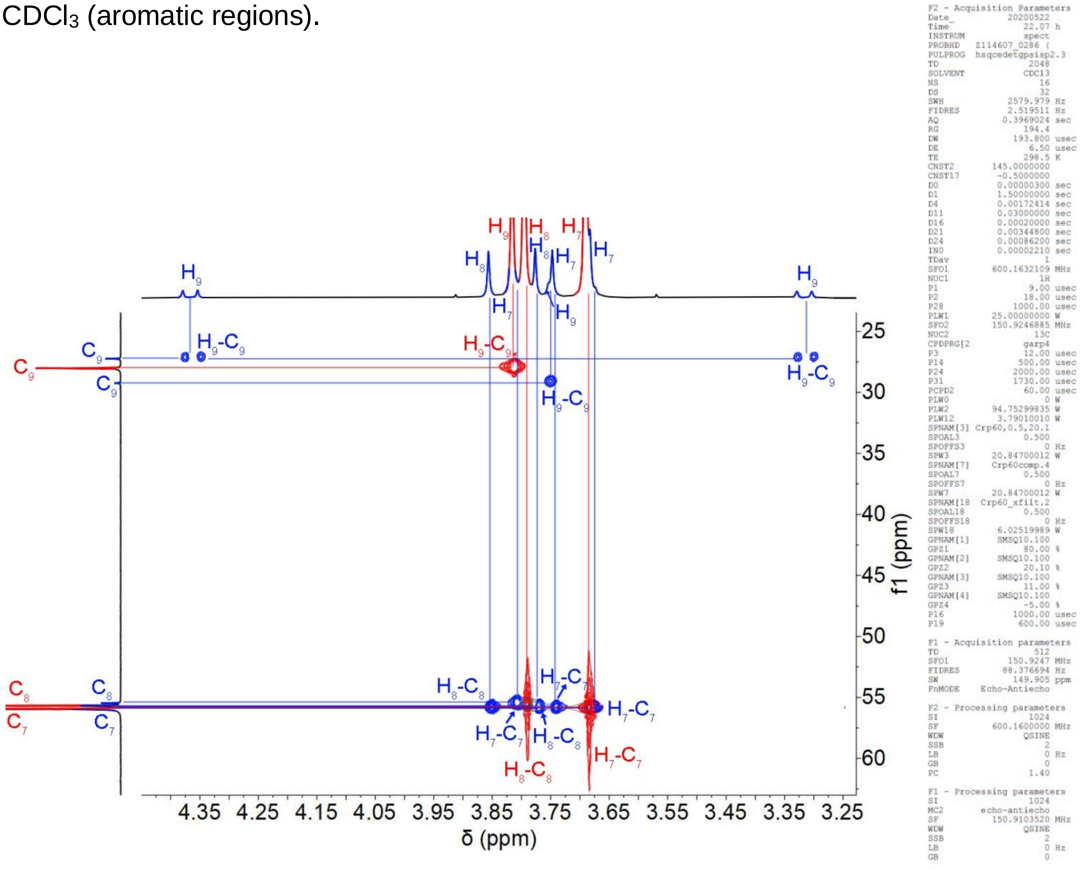

Figure S7 Partial ^{1}H-^{13}C HMQC NMR spectrum (600 MHz, 25 °C, 100 mM) of MeBP3 in $CDCl_3$ (methoxyl & methylene regions).

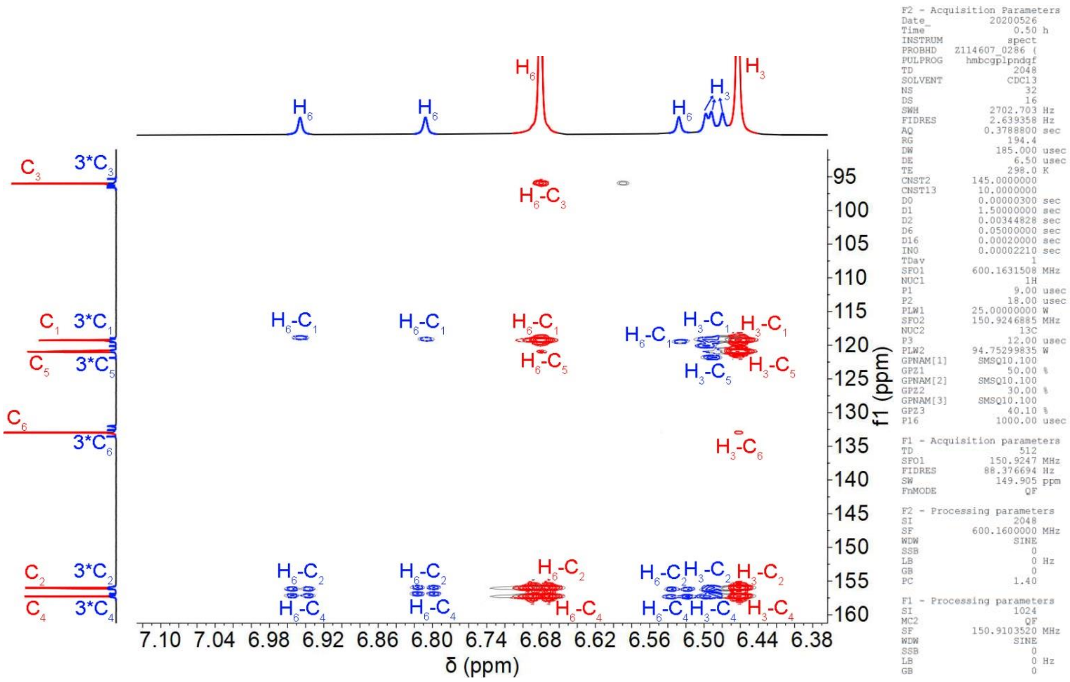

Figure S8 Partial ^{1}H-^{13}C HMBC NMR spectrum (600 MHz, 25 °C, 100 mM) of MeBP3 in $CDCl_3$ (aromatic regions to aromatic regions).

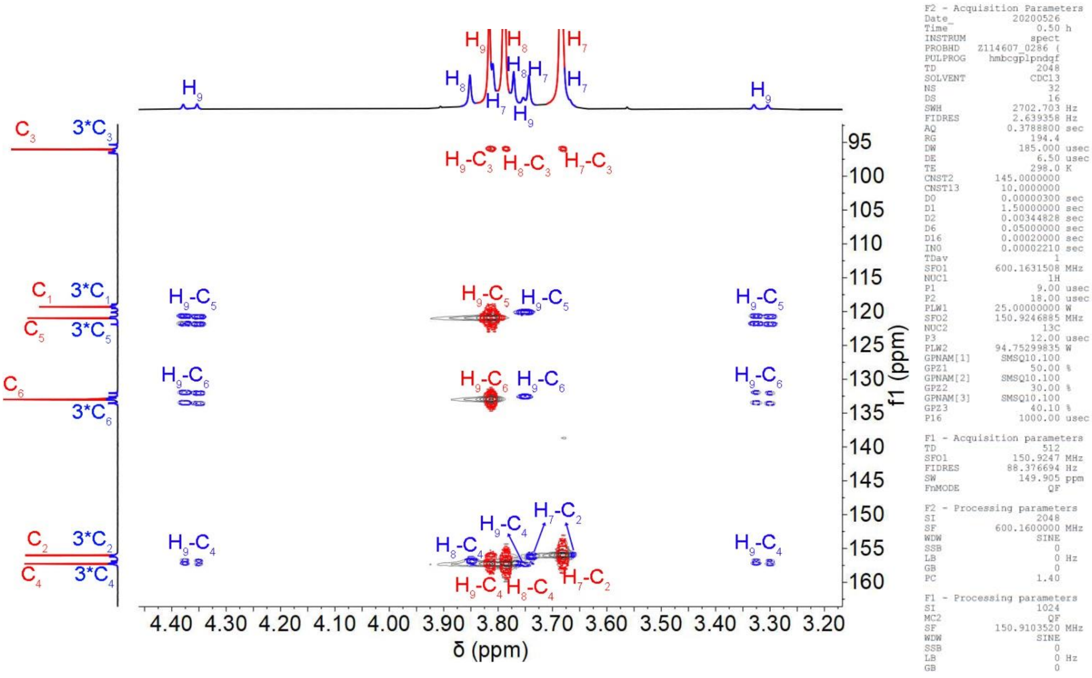

Figure S9 Partial ^{1}H-^{13}C HMBC NMR spectrum (600 MHz, 25 °C, 100 mM) of MeBP3 in $CDCl_3$ (aromatic regions to methoxyl-methylene regions).

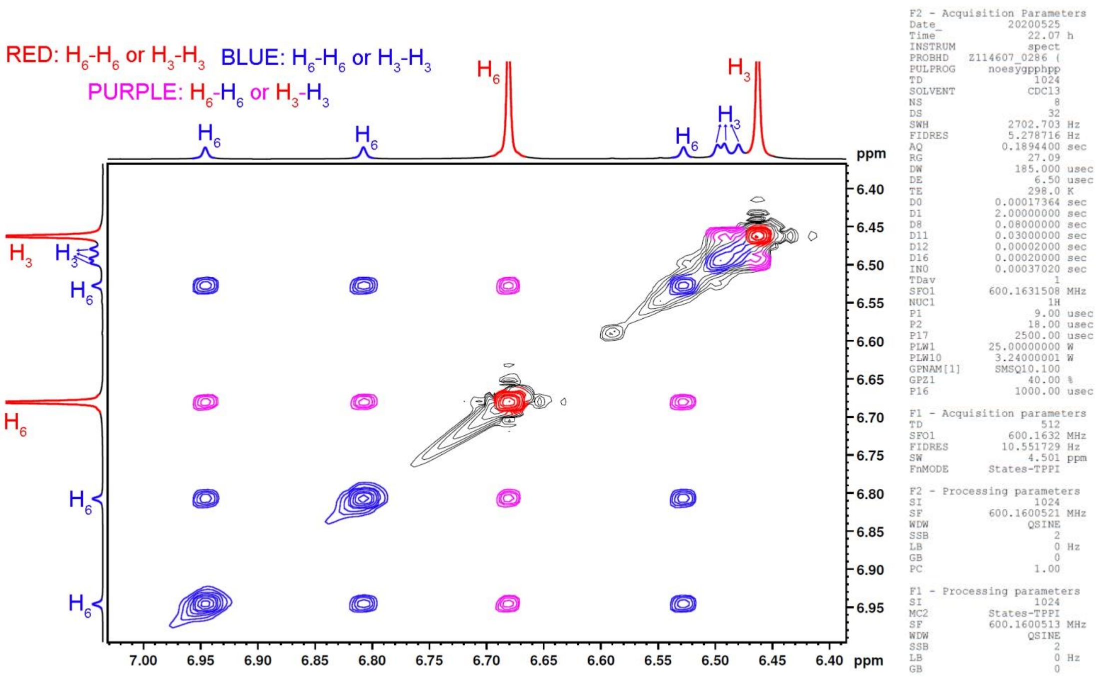

Figure S10. 2D NOESY (600 MHz, 298 K) spectrum of MeBP3 in $CDCl_3$ with a mixing time of 80 ms.

4. Copies of Variable-temperature ^{1}H NMR Spectra.

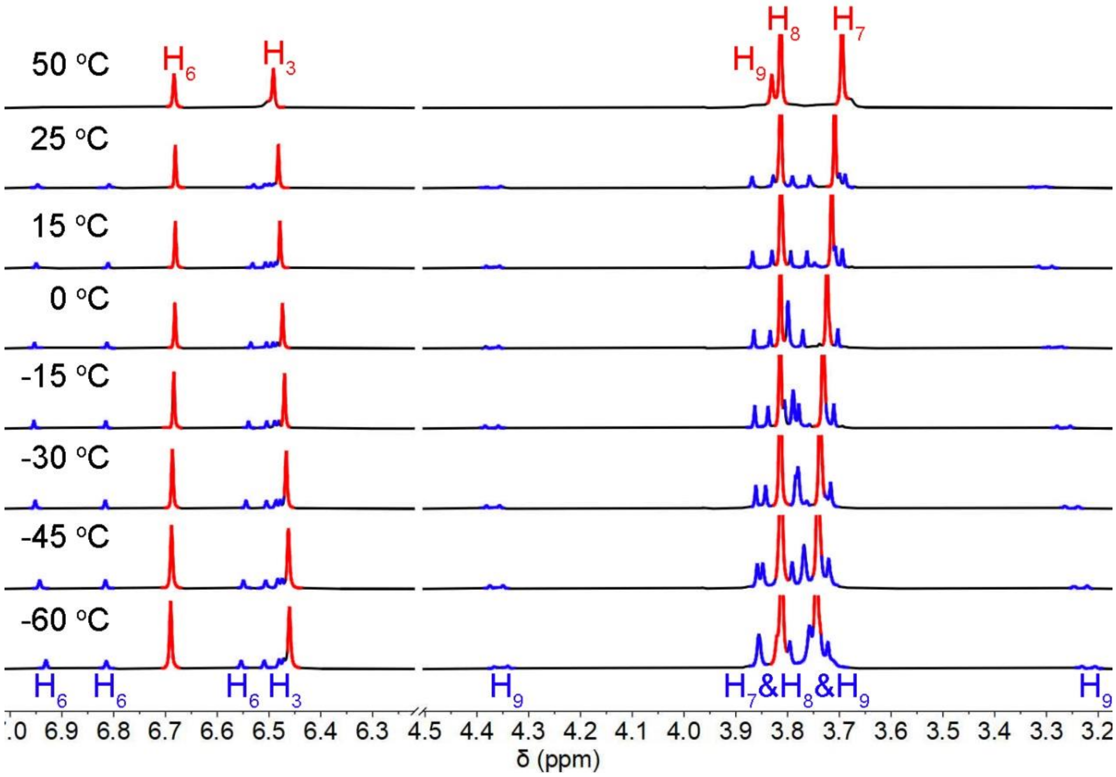

Figure S11 Variable-temperature ^{1}H NMR spectra of MeBP3 in $CDCl_3$ (400 MHz, 2.0 mM).

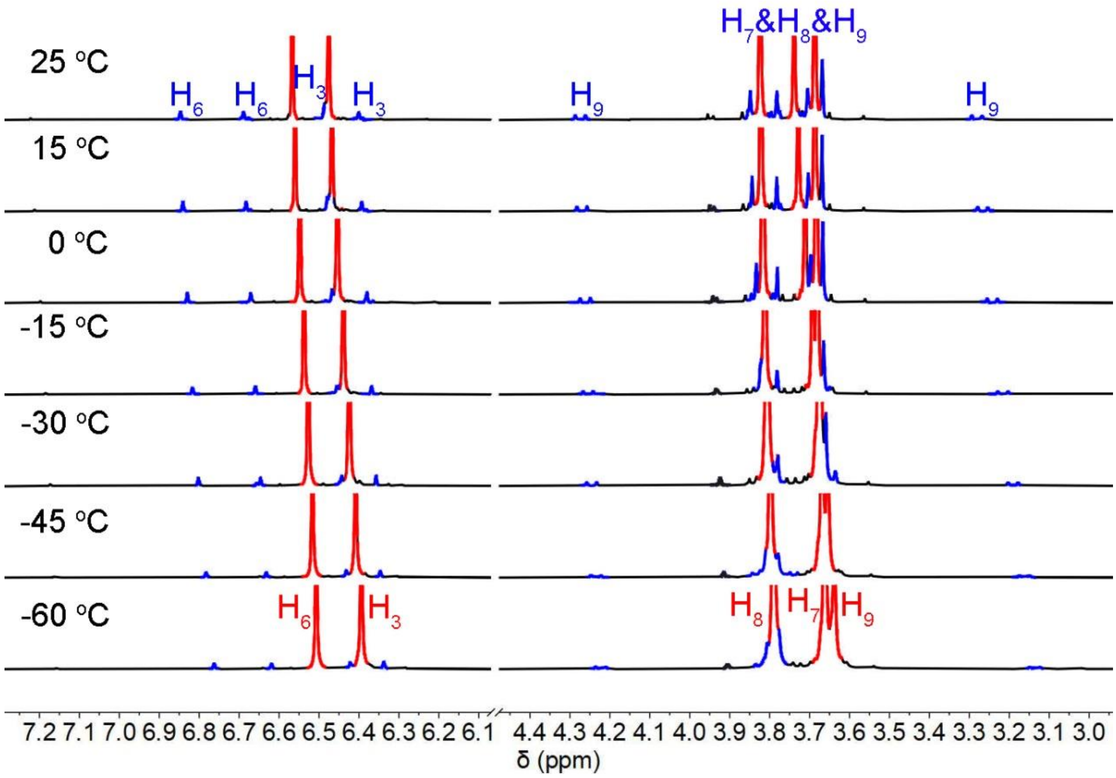

Figure S12 Variable-temperature ^{1}H NMR spectra of MeBP3 in CD_2Cl_2 (400 MHz, 2.0 mM).

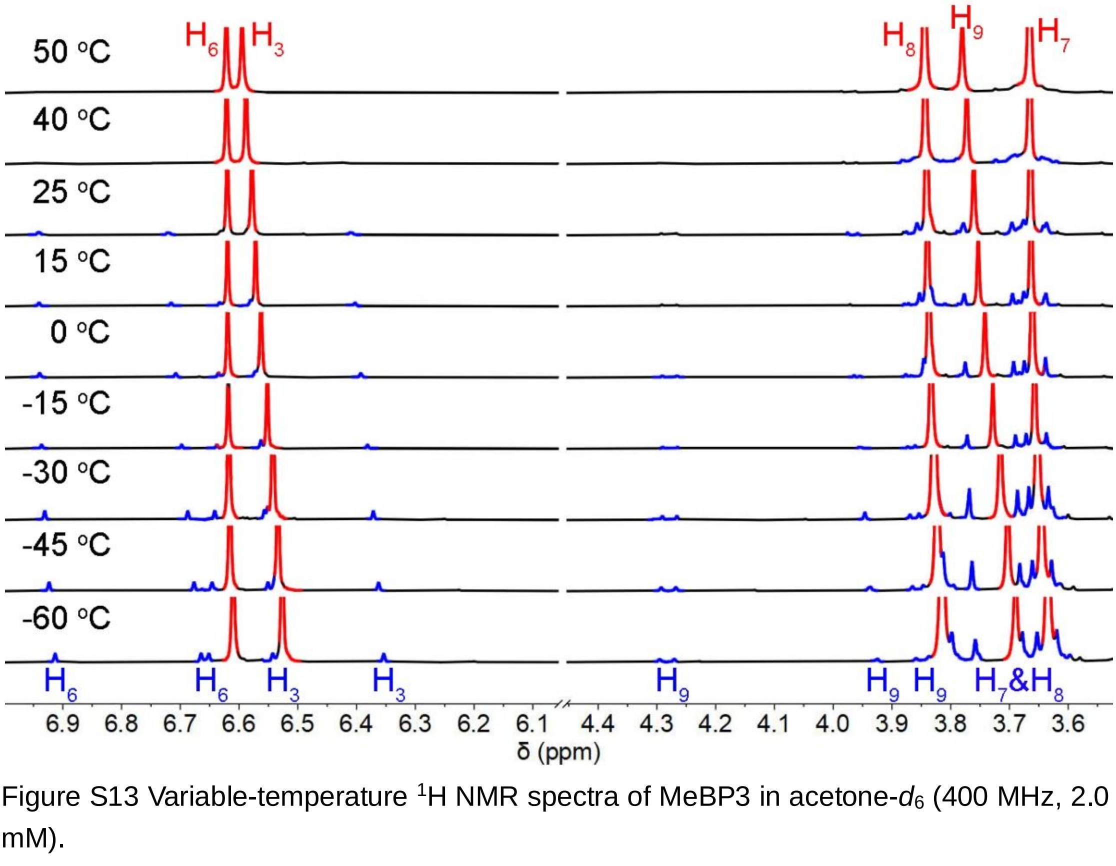

Figure S13 Variable-temperature ^{1}H NMR spectra of MeBP3 in acetone-d_6 (400 MHz, 2.0 mM).

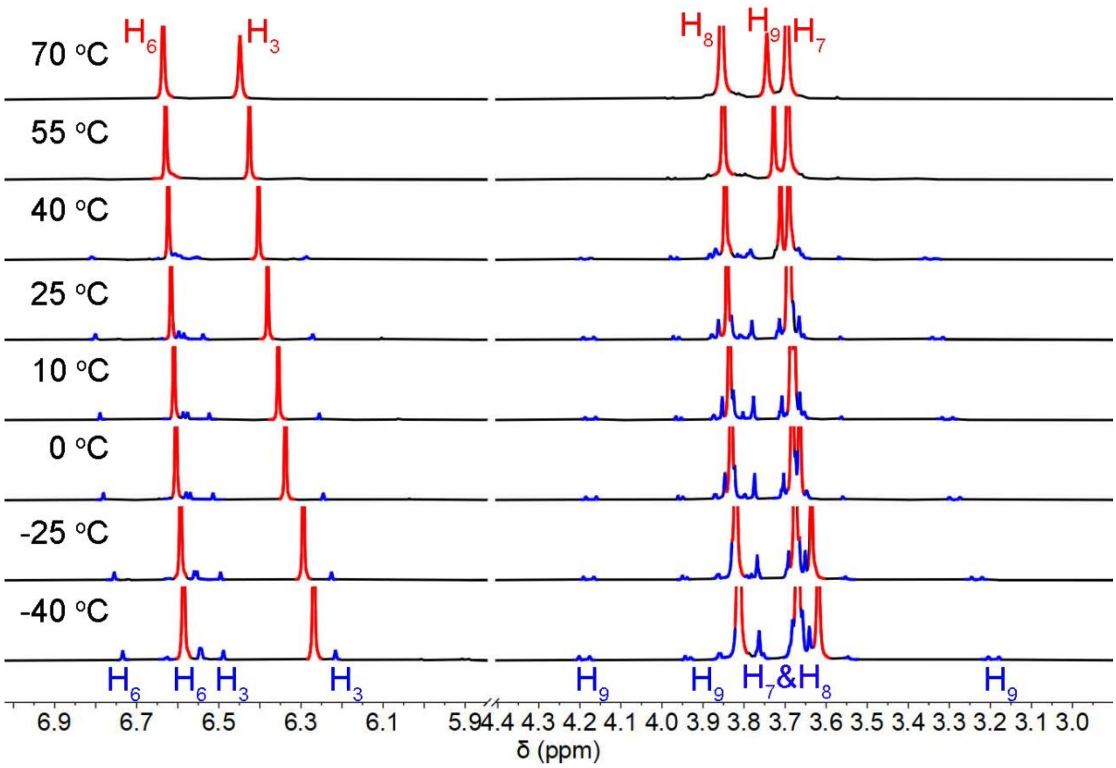

Figure S14 Variable-temperature ^{1}H NMR spectra of MeBP3 in acetonitrile-d_3 (400 MHz, 2.0 mM).

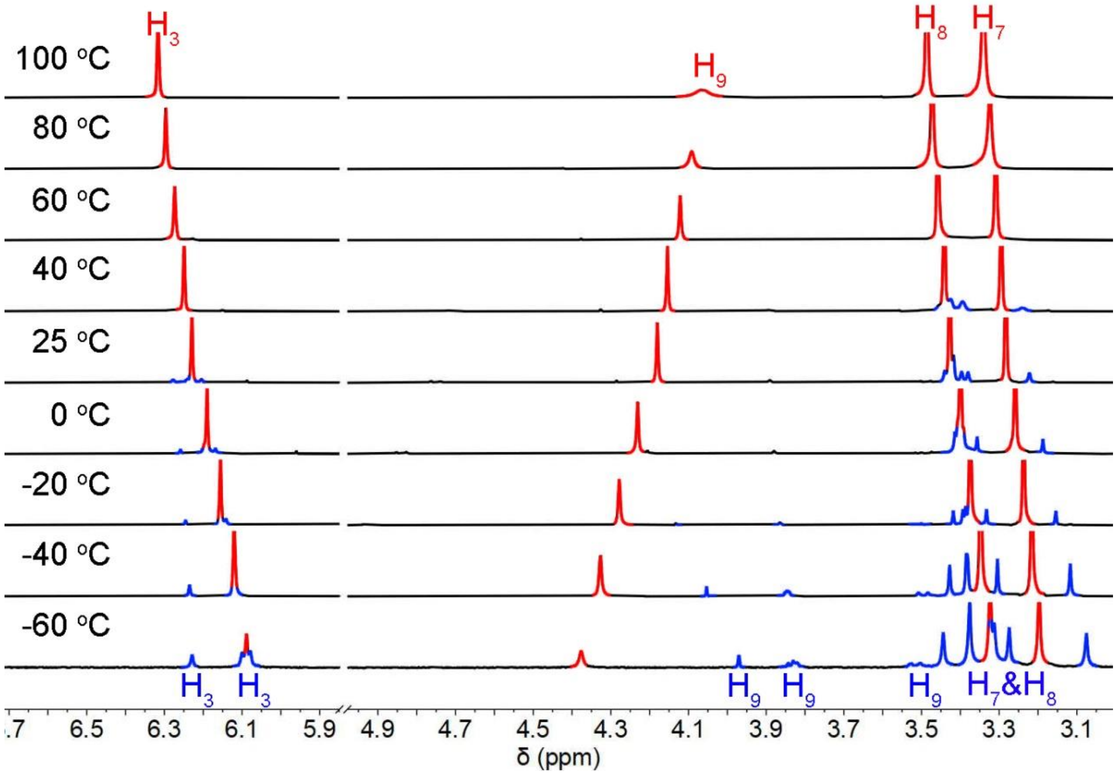

Figure S15 Variable-temperature ^{1}H NMR spectra of MeBP3 in toluene-d_8 (400 MHz, 2.0 mM).

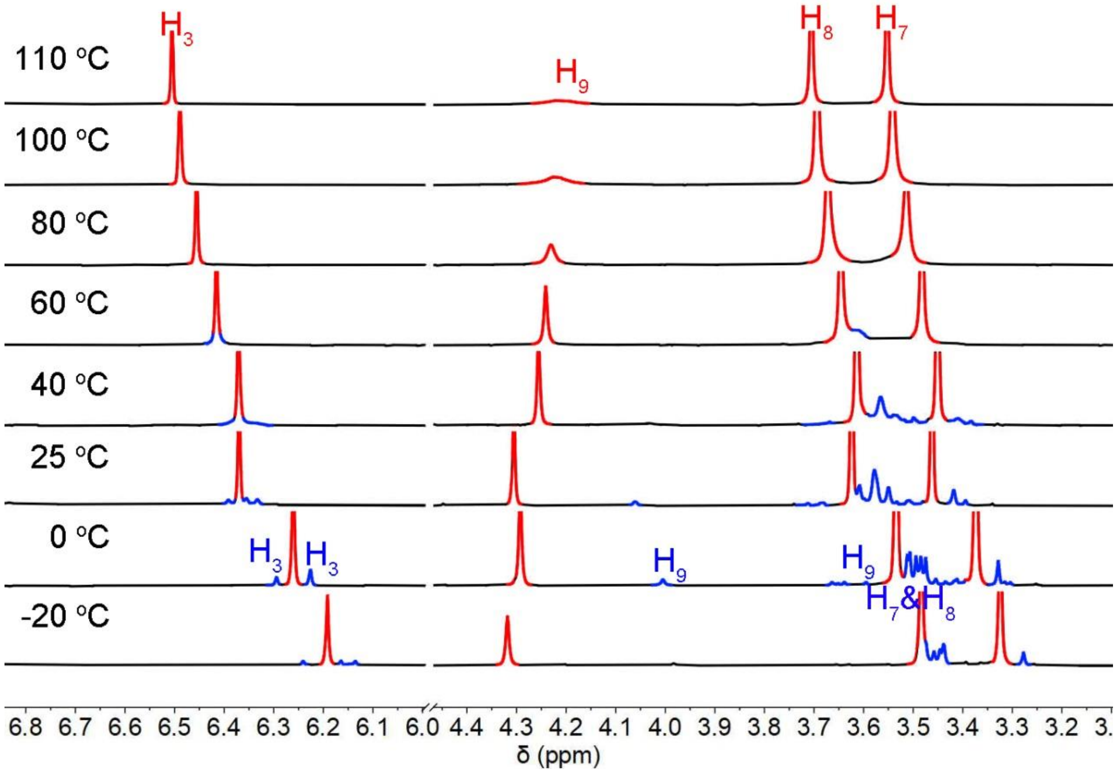

Figure S16 Variable-temperature ^{1}H NMR spectra of MeBP3 in xylene-d_{10} (400 MHz, 2.0 mM).

6. Tables of mole ratio of conformer I and conformer II ([I]/[II]).

Table S1 Percentages of MeBP3-**I** & MeBP3-**II** in different solvents and temperatures.

		$CDCl_3$	CD_2Cl_2	CD_3CN	acetone-d_6	xylene-d_{10}	toluene-d_8
-60[a]	MeBP3-I	74%	92%	-	88%	-	39%
	MeBP3-II	26%	8%	-	12%	-	61%
-45	MeBP3-I	72%	92%	-	88%	-	-
	MeBP3-II	28%	8%	-	12%	-	-
-40	MeBP3-I	-[b]	-	90%	-	-	73%
	MeBP3-II	-	-	10%	-	-	24%
-30	MeBP3-I	72%	92%	-	89%	-	-
	MeBP3-II	28%	8%	-	11%	-	-
-25	MeBP3-I	-	-	90%	-	-	-
	MeBP3-II	-	-	10%	-	-	-
-20	MeBP3-I	-	-	-	-	86%	85%
	MeBP3-II	-	-	-	-	14%	15%
-15	MeBP3-I	73%	91%	-	89%	-	-
	MeBP3-II	27%	9%	-	11%	-	-
0	MeBP3-I	74%	90%	90%	88%	85%	85%
	MeBP3-II	26%	10%	10%	12%	15%	15%
10	MeBP3-I	-	-	89%	-	-	-
	MeBP3-II	-	-	10%	-	-	-
15	MeBP3-I	74%	89%	-	88%	-	-
	MeBP3-II	26%	11%	-	12%	-	-
25	MeBP3-I	73%	87%	86%	88%	80%	85%
	MeBP3-II	27%	13%	14%	12%	20%	15%
40	MeBP3-I	-	-	87%	88%	85%	92%
	MeBP3-II	-	-	13%	12%	15%	8%
50	MeBP3-I	>95%	-	-	>95%	-	-
	MeBP3-II	<5%	-	-	<5%	-	-
55	MeBP3-I	-	-	>95%	-	-	-
	MeBP3-II	-	-	<5%	-	-	-
60	MeBP3-I	-	-	-	-	>95%	>95%
	MeBP3-II	-	-	-	-	<5%	<5%
70	MeBP3-I	-	-	>95%	-	-	-
	MeBP3-II	-	-	<5%	-	-	-
80	MeBP3-I	-	-	-	-	>95%	>95%
	MeBP3-II	-	-	-	-	<5%	<5%
100	MeBP3-I	-	-	-	-	>95%	>95%
	MeBP3-II	-	-	-	-	<5%	<5%
110	MeBP3-I	-	-	-	-	>95%	-
	MeBP3-II	-	-	-	-	<5%	-

[a]: ^{o}C; [b]: didn't test.

7. NMR Titration Experiment.

To determine the association constants of **1⁺** ⊂ MeBP3-I (K_1) and **1⁺** ⊂ MeBP3-II (K_2), NMR titrations were done with solutions which had a constant concentration of MeBP3 and varying concentrations of **1⁺**. Using the nonlinear curve-fitting method, the association constant was obtained for each host-guest combination from the following equations[3]:

$$\delta_{obs} = \frac{2(K_1 + KK_2)\delta_F + \delta_B K_1\{-(1 + K + (K_1 + KK_2)([H]_t - [G]_t)) + \sqrt{(1 + K + (K_1 + KK_2)([H]_t - [G]_t))^2 + 4(K_1 + KK_2)(1 + K)[G]_t}\}}{2(K_1 + KK_2) + K_1\{-(1 + K + (K_1 + KK_2)([H]_t - [G]_t)) + \sqrt{(1 + K + (K_1 + KK_2)([H]_t - [G]_t))^2 + 4(K_1 + KK_2)(1 + K)[G]_t}\}}$$

$$\delta_{obs} = \frac{2(K_1/K + K_2)\delta_F + \delta_B K_2\{-(1 + 1/K + (K_1/K + K_2)([H]_t - [G]_t)) + \sqrt{(1 + 1/K + (K_1/K + K_2)([H]_t - [G]_t))^2 + 4(K_1/K + K_2)(1 + 1/K)[G]_t}\}}{2(K_1/K + K_2) + K_2\{-(1 + 1/K + (K_1/K + K_2)([H]_t - [G]_t)) + \sqrt{(1 + 1/K + (K_1/K + K_2)([H]_t - [G]_t))^2 + 4(K_1/K + K_2)(1 + 1/K)[G]_t}\}}$$

Where $[H]_t$ and $[G]_t$ are the total concentration of the host and the guest, respectively; δ_{obs} is the chemical shifts of the proton of interest; δ_F and δ_B are the chemical shifts of the proton of interest in their free and bound states. The association constants was calculated by using the nonlinear curve-fitting method.

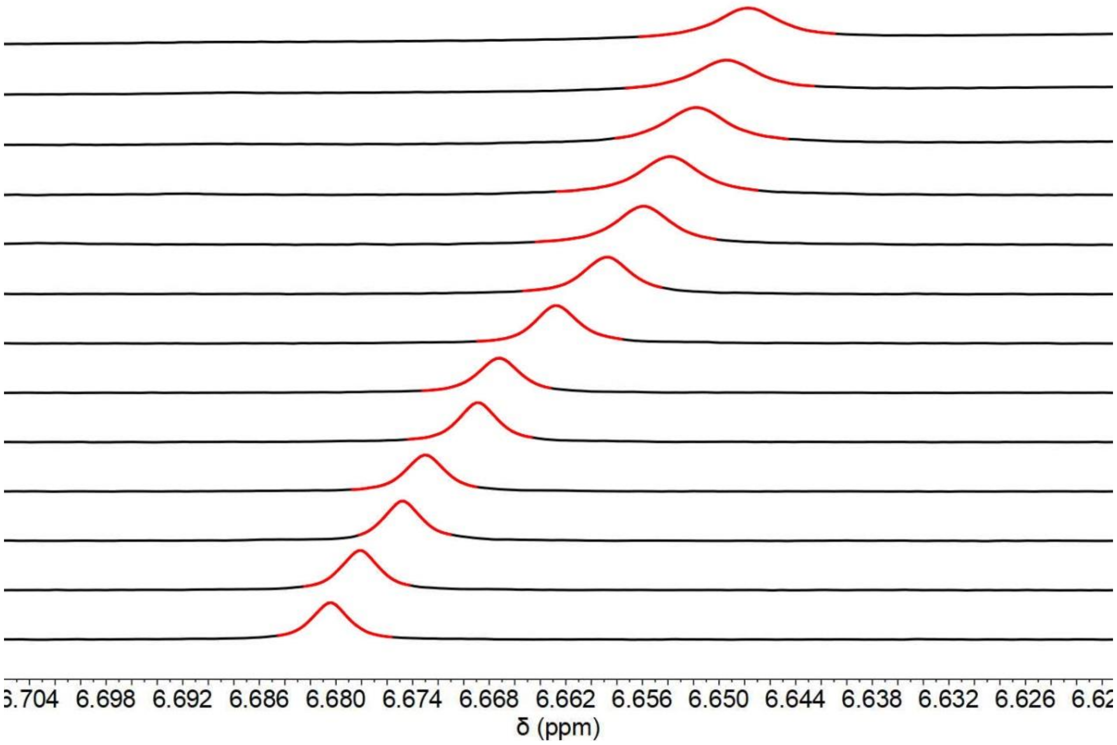

Figure S17 Partial ^{1}H NMR spectra (500 MHz, $CDCl_3$, 25 °C) of MeBP3 (H_6 of conformer I) at a concentration of 1.0 mM upon addition of **1⁺**. From bottom to top, the concentration of **1⁺** was 0.0, 0.1, 0.3, 0.4, 0.6, 0.8, 1.2, 2.0, 3.5, 5.8, 10.6 17.2 & 25.4 mM.

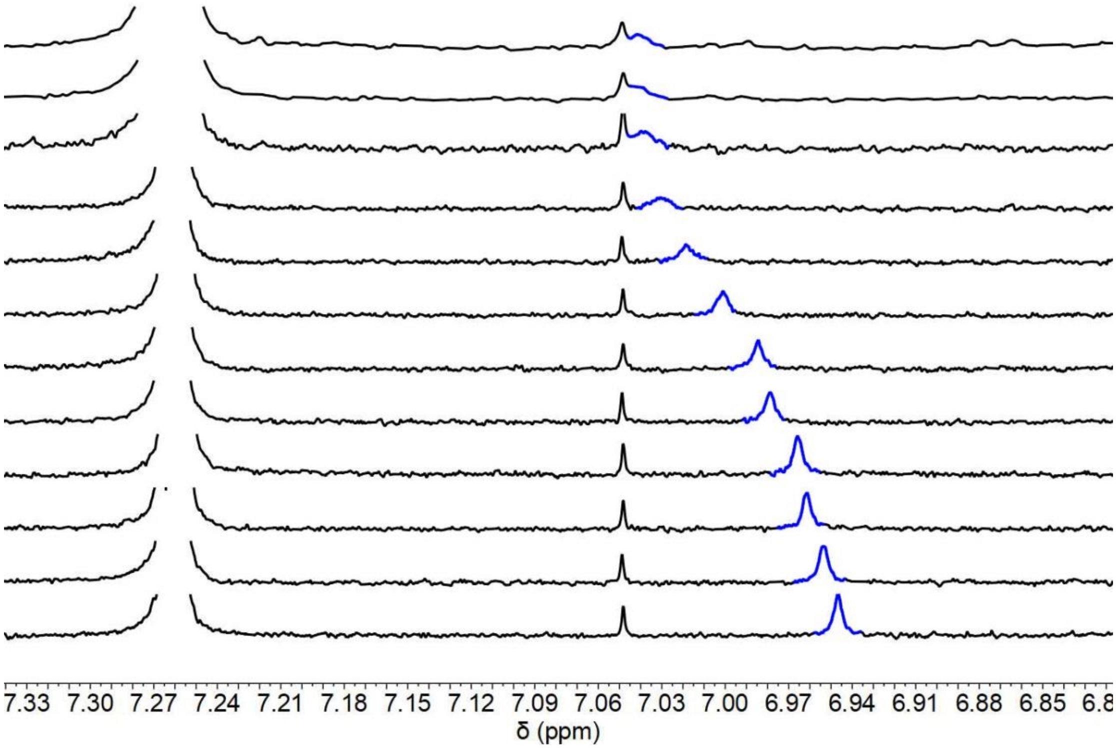

Figure S18 Partial ^{1}H NMR spectra (500 MHz, $CDCl_3$, 25 °C) of MeBP3 (H_6 of conformer **II**) at a concentration of 1.00 mM upon addition of **1⁺**. From bottom to top, the concentration of **1⁺** was 0.0, 0.1, 0.3, 0.4, 0.6, 0.8, 1.2, 2.0, 3.5, 5.8, 10.6 17.2 & 25.4 mM.

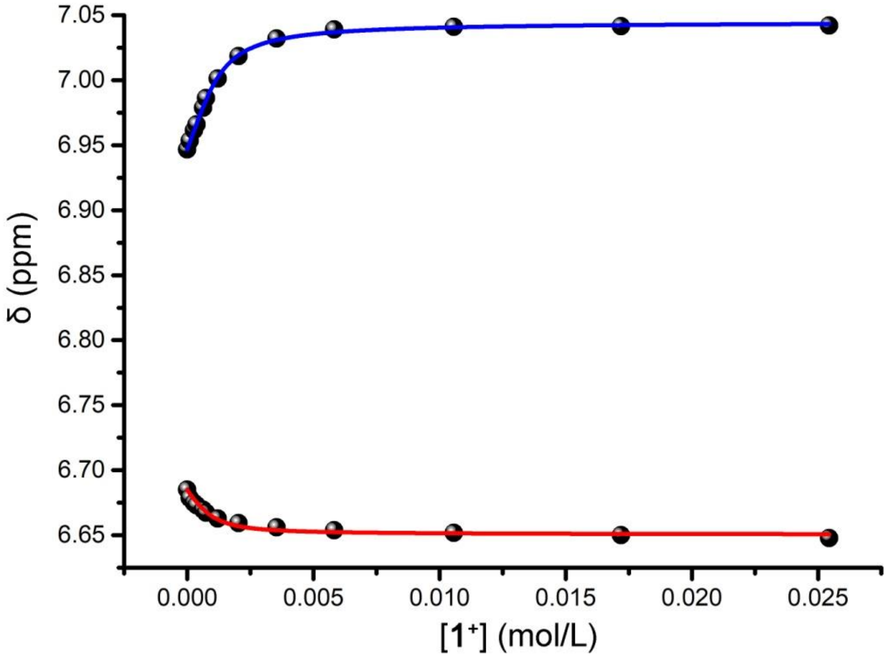

Figure S19 The non-linear curve-fitting (NMR titrations) for the complex of **1⁺** and conformer **I** & **II**, K_1 = (3.50 ± 0.54) × 10^3 M^{-1} and K_2 = (2.27 ± 0.18) × 10^3 M^{-1}.

8. References

(1) Y. Wang, K. Xu, B. Li, L. Cui, J. Li, X. Jia, H. Zhao, J. Fang and C. Li, *Angew. Chem. Int. Ed.* **2019**, *58*, 10281–10284.
(2) M. D. Rosa, C. Talotta, C. Gaeta, A. Soriente, P. Neri, S. Pappalardo, G. Gattuso, A. Notti, M. F. Parisi and I. Pisagatti, *J. Org. Chem.* **2017**, *82*, 5162–5168.
(3) L.-P.Yang, L. Zhang, M. Quan, J. S. Ward, Y.-L. Ma, H. Zhou, K. Rissanen and W. Jiang, *Nat. Commun.* **2020**, *11*: 2740.
